# A review of home-based physical activity interventions for breast cancer survivors

**DOI:** 10.25082/CCR.2019.01.002

**Published:** 2019-07-30

**Authors:** Steven S. Coughlin, Lee Caplan, Rebecca Stone, Jessica Stewart

**Affiliations:** 1 Department of Population Health Sciences, Medical College of Georgia, Augusta University, Augusta, GA 30912, USA; 2 Institute of Public & Preventive Health, Augusta University, Augusta, GA 30912, USA; 3 Morehouse School of Medicine, Department of Community Health and Preventive Medicine, Atlanta, GA 30310, USA; 4 Department of Interdisciplinary Health Sciences, College of Allied Health Sciences, Augusta University, Augusta, GA 30310, USA

**Keywords:** breast cancer survivors, physical activity, women

## Abstract

As breast cancer relative survival continues to increase, many breast cancer patients face many issues, including recurrence of cancer and cancer-related side effects that impact several aspects of their quality of life. With breast cancer patients living longer, there is more of a concern for negative breast cancer outcomes. Although physical activity is an affordable and relatively convenient way to improve breast cancer outcomes, only about one-third of breast cancer survivors engage in the recommended level of physical activity. This article reviews articles published to date to examine whether home-based physical activity interventions are effective in improving physical activity and other outcomes among breast cancer survivors who have completed primary therapy for the disease. The present review is based upon bibliographic searches in PubMed and CINAHL and relevant search terms. Articles published in English from 1980 through February 28, 2019 were identified. A total of 360 article citations were identified in PubMed and non-duplicates in CINAHL. After screening the abstracts or full texts of these articles and reviewing the references of previous review articles, 20 studies that met the eligibility criteria. Three of the studies were pre-/post-test trials and 17 were randomized controlled trials. home-based exercise programs are effective in improving physical activity among breast cancer survivors who have completed primary therapy for the disease. Home-based exercise programs such as walking programs offer a convenient and affordable option for women who wish to increase their physical activity and maintain a healthy lifestyle.

## 1 Introduction

The five-year relative breast cancer survival rate in the US continues to increase and is now about 91%^[1]^. As the rate increases, many breast cancer patients face many issues, including recurrence of cancer and cancer-related side effects that impact several aspects of their quality of life^[2]^. To reduce risk of cancer recurrence, the American Institute for Cancer Research also recommends that cancer survivors meet physical activity guidelines (AICR)^[3]^. Of women diagnosed with breast cancer, 50–96% experience weight gain during treatment^[4]^. This weight gain after diagnosis usually ranges between 2.5 and 6.2 kg (5.5 to 13.6 lbs)^[5]^. Among breast cancer survivors (BCSs), physical activity improves physical functioning, cardiovascular fitness, emotional wellbeing, and psychological adjustment, while lowering fatigue, depression, and anxiety, and helping to maintain a healthy body weight^[6]^. In addition, studies suggest that the immunological status of breast cancer patients improves after physical activity^[7]^. However, levels of physical activity in this population are low.

Physical inactivity and excessive weight gain that can occur following breast cancer treatment increases the risk of breast cancer recurrence, other chronic diseases, and all-cause and breast cancer-related mortality^[8]^. Physical inactivity increases the risk of obesity, which increases circulating estrogen levels and mortality. Exercise can lower circulating levels of estrogen and potentially reduce tumor proliferation. Although physical activity is an affordable and relatively convenient way to improve breast cancer outcomes, only about one-third of breast cancer survivors engage in the recommended level of physical activity^[9]^.

Home-based exercise programs, including walking programs, offer a convenient and affordable option for women who wish to increase their physical activity and maintain a healthy lifestyle. This manuscript reviews articles published to date to examine whether home-based physical activity interventions are effective in increasing physical activity and improving other outcomes among breast cancer survivors who have completed primary therapy for the disease (adjuvant chemotherapy, radiation, or surgery).

## 2 Methods

The present review is based upon bibliographic searches in PubMed and CINAHL (Cummulative Index to Nursing and Allied Health Literature) and relevant search terms. Articles published in English from 1980 through February 28, 2019 were identified using the following MeSH (Medical Subject Heading) search terms and Boolean algebra commands: home based AND physical activity AND breast cancer. The following MeSH search terms and Boolean algebra commands were also used: walking intervention AND breast cancer. The searches were not limited to words appearing in the title of an article nor to studies in a particular country or geographic region of the world. The references of review articles were also reviewed (Bluethman *et al*. 2015; Paxton *et al.* 2019). Information obtained from bibliographic searches (title and topic of article, information in abstract, study design, and key words) was used to determine whether or not to retain each identified article. Only studies written in English that examined the impact of breast cancer survivorship care plans on health outcomes were eligible for inclusion.

## 3 Results

**Figure 1.**
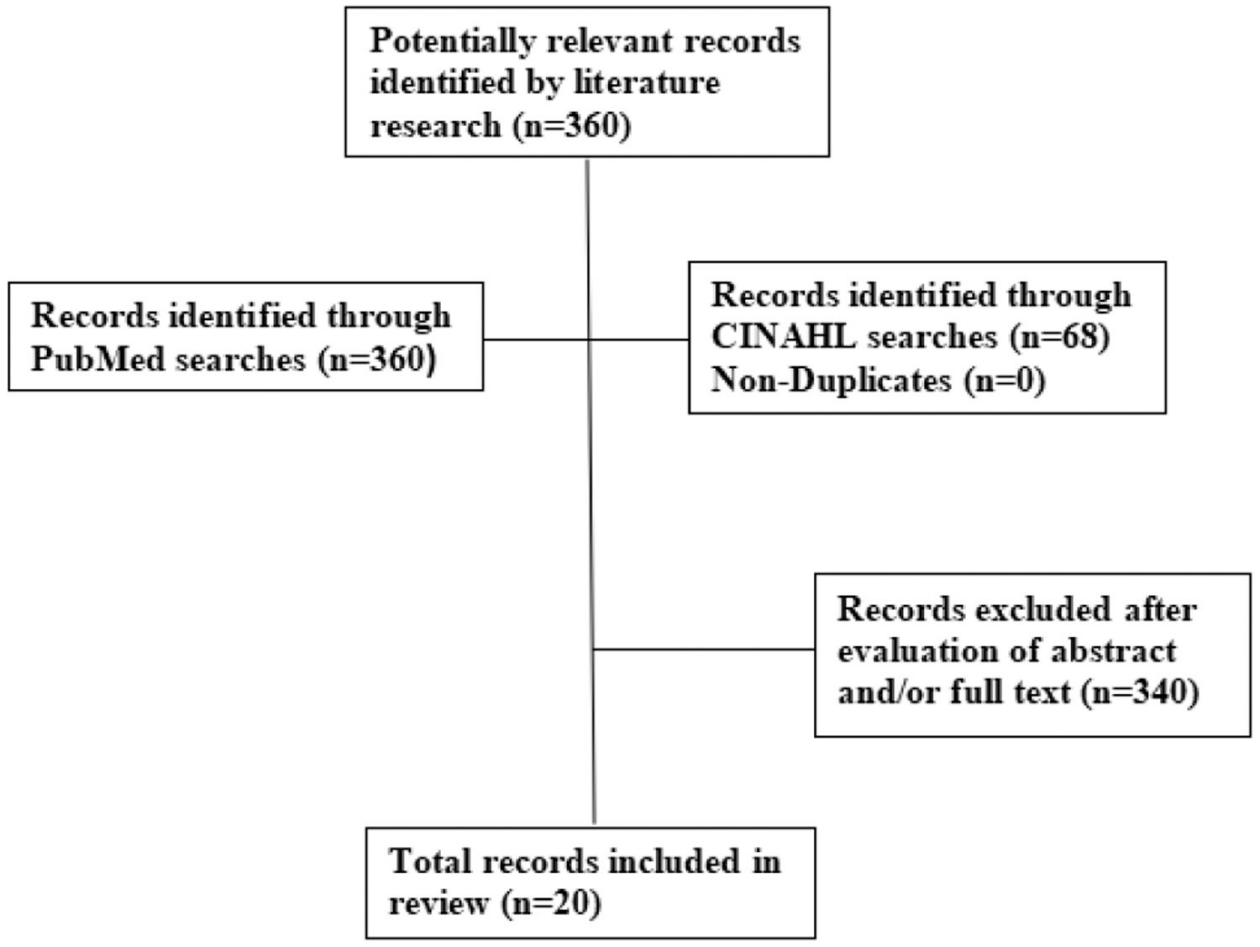
Flowchart of record selection process

A total of 360 article citations were identified in PubMed and non-duplicates in CINAHL (Figure 1). After screening the abstracts or full texts of these articles and reviewing the references of previous review articles, we were left with 20 studies that met the eligibility criteria. Three of the studies were pre-/post-test trials, and 17 were randomized controlled trials.

Pinto *et al*.^[10]^ conducted a 12-week randomized controlled trial of physical activity counseling delivered via telephone, combined with weekly exercise tip sheets (Table 1). Eighty-six women who had completed therapy for stage 0-II breast cancer were enrolled in the trial. The physical activity group reported significantly more total minutes of physical activity and more minutes of moderate-intensity physical activity than the control group (p = 0.001).

In an eight-week pre/post-test trial of a community intervention that combined the use of pedometers with scheduling, goal setting, and self-assessment, Wilson *et al*.^[11]^ found that the intervention led to significant increases in steps walked per day and improved attitude toward exercise, as well as significant decreases in body mass index and other anthropometric measures. Twenty-four African American breast cancer survivors were enrolled in the trial.

Vallance *et al*.^[12]^ conducted a 12-week randomized controlled trial with four arms: i) standard public health recommendation for physical activity; ii) breast cancer-specific physical activity print materials; iii) use of a step pedometer; or iv) a combination of print materials and use of a step pedometer. Physical activity increased by three minutes/week in the standard recommendation group compared with 70 minutes/week in the print material group (p = 0.117), 89 minutes/week in the pedometer group (p = 0.017), and 87 minutes/week in the combined group (p = 0.022). For brisk walking, all three intervention groups reported significantly greater increases than the standard recommendation group. The combined group also reported significantly improved quality-of-life (p = 0.003) and reduced fatigue (p = 0.052).

In a 12-week randomized controlled trial of a home-based walking intervention, Matthews *et al*.^[13]^ found that intervention participants reported a significantly greater increase in walking for exercise than the controls (p = 0.01). In a 12-week randomized controlled trial of two home-based exercise programs (aerobic exercise, resistance training), Yuen and Sword^[14]^ found a significant reduction in fatigue among participants in the aerobic exercise group compared with the resistance exercise group (p = 0.006). In addition, there was a significant improvement in the distance walked during a 6-minute walk test in the resistance exercise group (p = 0.009).

Payne *et al*.^[15]^ conducted a randomized controlled trial of a home-based walking program among 20 women receiving hormonal therapy for breast cancer. There was a significant improvement in sleep scores in the intervention group compared with the control group (p = 0.007). Serotonin levels were also significantly different between groups (p = 0.009).

Yang *et al*.^[16]^ conducted a 12-week randomized controlled trial of a home-based walking program among 40 breast cancer patients who were receiving an aromatase inhibitor. Women in the exercise group reported significantly lower symptom severity scores (p<0.01) and mood disturbance (p = 0.02) compared with those in the control group.

In a 12-week randomized controlled trial of a home-based, stage-matched, exercise and diet intervention (telephone counseling and a workbook), Kim *et al*.^[17]^ found that the intervention group showed significantly greater improvement in emotional functioning (p = 0.04), and motivational readiness for exercise (p<0.006) and dieting (p<0.001), and reduced fatigue (p = 0.001) and depression (p = 0.035) than the controls.

In a 12-week randomized controlled trial of an email physical activity intervention, Hatchett *et al*.^[18]^ observed significantly higher levels of physical activity in the intervention group at six (p = 0.001) and 12 weeks (p<0.001) compared to the controls.

Spector *et al*.^[19]^ conducted a pre/post-test trial of a home-based aerobic and resistance training exercise intervention. The intervention included motivational interviewing and weekly telephone calls. They found a significant increase in total minutes of weekly physical activity (p = 0.001). Total quality-of-life and fatigue scores improved, but neither improvement was statistically significant.

Denysschen *et al*.^[20]^ conducted an eight-week pre/post-test trial of a home-based exercise program (resistance exercises and self-selected aerobic exercise) among 26 breast cancer patients who were receiving an aromatase inhibitor. The participants reported a significantly lower number of painful joints and significantly improved quality-of-life (p<0.05). Significant improvements in grip strength (p<0.01), biceps curl (p<0.01), and sit-to-stand were also observed. There were no significant differences in anthropometric measures or cardiovascular endurance.

In a 12-week randomized controlled trial of a home-based walking program among 32 early-stage breast cancer survivors, Baruth *et al*.^[21]^ found that participants in the intervention group had reduced fatigue and improvement in other quality of life outcomes.

Lahart *et al*.^[22]^ conducted a six-month randomized controlled trial of a physical activity intervention (face-to-face and telephone physical activity counseling) among 80 breast cancer survivors. Total, leisure, and vigorous physical activity increased in the intervention group compared to the usual care group (p = 0.24, p = 0.01, and p = 0.007, respectively). Both body mass and body mass index decreased significantly in the intervention group compared to the usual care group (p = 0.04 and p = 0.02, respectively). In addition, total cholesterol and LDL-cholesterol decreased significantly in the intervention group (p = 0.001) but not in the usual care group (p = 0.23).

Knobf *et al*.^[23]^ conducted a 12-month randomized controlled trial of an aerobic-resistance exercise intervention compared to a home-based physical activity intervention among 154 early postmenopausal breast cancer survivors. The outcomes of interest were bone mineral density and biomarkers of bone turnover. No significant difference in bone mineral density was observed between the two groups.

Nyrop *et al*.^[24]^ conducted a six-week randomized controlled trial of a home-based walking program among 62 post-menopausal breast cancer patients with aromatase inhibitor-associated arthralgia. Intervention group participants reported significantly increased walking minutes per week, reduced stiffness, less difficulty with activities of daily living, and less perceived helplessness in managing joint symptoms compared to the controls.

Valle *et al*.^[25]^ conducted a two-arm, six-month randomized controlled trial of a physical activity intervention (activity tracker and tailored feedback based on objective weight; tailored feedback alone; or control). The outcome of interest was change in weight. Thirty-five African American breast cancer survivors participated in the trial. Median weight change was −0.9% in the intervention group that included activity trackers vs. 0.2% gain in the control group.

Westphal *et al*.^[26]^ conducted a 48-week, multi-center, randomized controlled trial of counseling and unsupervised exercise training vs. supervised physical training (24 weeks) followed by unsupervised training (additional 24 weeks). The supervised training was comprised of 45 minutes of stationary cycling and 30 minutes of resistance training twice a week. After 24 weeks, the supervised arm achieved a significantly higher maximum output in watt (132 ± 34, 95% CI: 117-147) compared to baseline 107 ± 25, 95% CI: 97-117, p = 0.012). In addition, output was higher in the supervised arm (115 ± 25, 95% CI: 105-125, p = 0.059) than in the unsupervised arm.

Hirschey *et al*.^[27]^ conducted a randomized controlled trial of a home-based physical activity intervention (a booklet about physical activity for breast cancer survivors, that included narrative messages and writing and thinking exercises) among 60 breast cancer survivors. Subjective exercise (weekly minutes) increased by two minutes, and objective exercise increased by 970 steps every four weeks in the intervention group compared to the control group (p = 0.2676 and p = 0.0283, respectively).

Bail *et al*.^[28]^ conducted a 12-month randomized controlled trial of a home-based mentored vegetable gardening intervention among 82 breast cancer survivors. Compared with the controls, intervention participants reported significantly greater improvements in moderate physical activity and demonstrated improvements in the two-minute step test and arm curl (p < 0.05).

Lahart *et al*.^[29]^ conducted a six-month randomized controlled trial of a home-based physical activity intervention involving 32 breast cancer survivors. Magnitude-based inference analyses revealed at least small beneficial effects on absolute and relative VȮ_2_ max (cardiorespiratory fitness), and total and moderate physical activity in the intervention compared to the usual care group.

## 4 Discussion

The results of this systematic literature review indicate that home-based exercise programs are effective in improving physical activity among breast cancer survivors who have completed primary therapy for the disease. A variety of outcomes were assessed in the trials, including self-reported minutes of physical activity, steps walked per day, functional capacity, cardiovascular endurance, body mass index, weight, sleep scores, quality-of-life, and attitudes toward physical activity. In the one trial that assessed bone mineral density as an outcome^[6]^, no significant difference was observed between the two groups. A variety of intervention strategies have been tested in trials of home-based exercise programs, including aerobic and resistance exercise training, walking programs, print materials, telephone counseling, and gardening. A majority of the outcomes measured in the trials using one or more of the intervention strategies demonstrated positive change in the intervention groups compared to the controls. These studies yielded encouraging information and reflected the acceptance of innovative methods of physical activity interventions by breast cancer survivors.

Three studies of the effectiveness of home-based exercise programs focused on African American breast cancer survivors^[11,19,25]^. The results of these studies indicated that home-based exercise programs are effective in increasing physical activity and reducing weight among African American breast cancer survivors.

Four studies of the effectiveness of home-based exercise programs focused on women receiving aromatase inhibitor therapy for breast cancer^[20,24,26]^. The results of these studies indicated that home-based exercise programs are effective in reducing joint pain and increasing mobility among women receiving this therapy.

Two RCTs focused on using an email physical activity intervention among breast cancer survivors^[17,18]^. Both studies were effective using email, showing improvement with motivation readiness for exercise, diet, emotional functioning, depression and significant differences in physical activity.

Four studies on the effectiveness of home-based walking interventions focused on breast cancer survivors, with one of the groups in each study receiving hormonal therapy^[13-15,21]^. These studies demonstrated that home-based walking interventions are effective in increasing walking for exercise, reducing fatigue, and improving quality of life. Those receiving hormonal therapy reported improvement in sleep scores and increased serotonin levels.

With respect to limitations, outcomes, intervention strategies, and tests varied among the studies. Caution is therefore required in comparing results across studies. In addition, our literature review may not have captured all relevant studies.

In summary, home-based exercise programs are effective in improving physical activity among breast cancer survivors who have completed primary therapy for the disease. This includes women who are receiving aromatase inhibitor therapy for breast cancer. Home-based exercise programs, such as walking programs offer a convenient and affordable option for women who wish to increase their physical activity and maintain a healthy lifestyle.

## Figures and Tables

**Table 1.** Studies of home-based physical activity interventions for breast cancer survivors

Author	Design	Outcomes	Sample Size	Results
Pinto et al., 2005	12-week randomized controlled trial of physical activity counseling delivered via telephone, combined with weekly exercise tip sheets	Self-reported minutes of physical activity	86 women who had completed therapy for stage 0-II breast cancer	The physical activity group reported significantly more total minutes of physical activity and more minutes of moderate-intensity physical activity (p=0.001)
Wilson et al.,2005	8-week pre-/ post-test trial of a community intervention using pedometers with scheduling, goal setting, and self-assessment	Steps walked per day, body mass index, and attitudes	24 African American breast cancer survivors	Significant increases in steps walked per day and attitude toward exercise were reported, as well as significant decreases in body mass index and other anthropometric measures
Vallance et al.,2007	12-week randomized controlled trial with 4 arms: standard public health recommendation for physical activity; breast cancer-specific physical activity print materials; a step pedometer; or a combination of print materials and step pedometers	Self-reported moderate/vigorous physical activity minutes per week. Secondary outcomes were quality-of-life, fatigue, self-reported brisk walking, and objective step counts	377 women who had completed therapy for stage I-IIIa breast cancer	Physical activity increased by 3- minutes/week in the standard recommendation group compared with 70 minutes/week in the print material group (p=0.117, 89 minutes/week in the pedometer group (p=0.017), and 87 minutes/week in the combined group (p=0.022). For brisk walking, all three intervention groups reported significantly greater increases than the standard recommendation group. The combined group also reported significantly improved quality-of-life (p=0.003) and reduced fatigue (p=0.052).
Matthews et al., 2007	12-week randomized controlled trial of a home-based walking intervention	Self-reported physical activity	36 breast cancer survivors	Intervention participants reported a significantly greater increase in walking for exercise (p=0.01)
Yuen & Sword, 2007	12-week randomized controlled trial of two home-based exercise programs (aerobic exercise, resistance training)	Fatigue, functional capacity (6-minute walk test)		There was a significant reduction in fatigue among participants in the aerobic exercise group (p=0.006). There was a significant improvement in the distance of the 6-minute walk test in the resistance exercise group (p=0.009)
Payne et al., 2008	Randomized control trial of a home-based walking program	Fatigue, sleep disturbances, depressive symptoms, and biomarkers (cortisol, serotonin, interleukin-6, bilirubin)	20 women receiving hormonal therapy for breast cancer	There was a significant improvement in sleep scores in the intervention group compared with the control group (p=0.007). Serotonin levels were also significantly different between groups (p=0.009)
Yang et al., 2010	12-week randomized controlled trial of a home-based walking program	Symptom severity score and mood disturbance	40 breast cancer patients (stage I-IIIa) receiving an aromatase inhibitor	Women in the exercise group reported significantly lower symptom severity scores (p<0.01) and mood disturbance (p=0.02) compared with those in the control group
Kim et al. 2011	12-week randomized controlled trial of a home-based stage-matched exercise and diet intervention (telephone counseling and a workbook	Stage of motivational readiness for exercise and diet, physical activity, diet quality, quality-of-life, fatigue, anxiety, depression	45 breast cancer survivors, stage 0-III	The intervention group showed significantly greater improvement in motivational readiness for exercise (p<0.006) and diet (p<0.001), emotional functioning (p=004), fatigue (p=0.001), and depression (p=0.035)
Hatchett et al.,2013	12-week randomized controlled trial of an email physical activity intervention	Self-reported physical activity	74 breast cancer survivors	Significant differences in levels of physical activity were observed between groups at 6 (p=0.001) and 12 weeks (p<0.001)
Spector et al.,2014	Pre-/post-test trial of a home-based aerobic and resistance training exercise intervention (motivational interviewing and weekly telephone calls)	Self-reported and objectively assessed physical activity, quality-of-life, and fatigue	17 African American women who had completed therapy for stage 0-IIIa breast cancer, who were currently sedentary	There was a significant increase in total minutes of weekly physical activity (p=0.001). Total quality-of-life and fatigue scores improved, but neither was significant.
Denysschen et al., 2014	8-week pre-/ post-test trial of a home-based exercise program (resistance exercises and self-selected aerobic exercise)	Anthropometry and functional performance and cardiovascular endurance (3-minute step test)	26 breast cancer patients receiving an aromatase inhibitor	Participants reported a significantly lower number of painful joints, and improved quality-of-life (p<0.05). Significant improvements in grip strength (p<0.01), biceps curl (p<0.01), and sit-to-stand were also observed. There were no significant differences in anthropometric measures or cardiovascular endurance.
Baruth et al.,2015	12-week randomized controlled trial of a home-based walking program	Self-reported fatigue, quality-of-life, and walking	32 women who were early stage breast cancer survivors	Participants in the intervention group had improvements in fatigue and quality of life outcomes. Changes in fatigue and quality of life were associated with changes in walking behavior
Lahart et al., 2016	6-month randomized controlled trial of a physical activity intervention (face-to-face and telephone physical activity counseling)	Physical activity (primary outcome) and body mass, body mass index, body fat, health-related quality-of-life, insulin resistance, and lipids	80 post-adjuvant therapy breast cancer patients	Total, leisure and vigorous physical activity significantly increased in the intervention group compared to usual care group (p= 0.24, p=0.01, and p=0.007, respectively). Both body mass and body mass index decreased significantly in the intervention group compared to usual care group (p=0.04 and p=0.02, respectively). Total cholesterol and LDL-cholesterol decreased significantly in the intervention group compared to usual care group (p=0.001 and p=0.23, respectively).
Knobf et al., 2016	12-month randomized controlled trial of a aerobic-resistance exercise intervention compared to a home-based physical activity intervention	Bone mineral density and biomarkers of bone turnover	154 early postmenopausal breast cancer survivors	No significant difference in bone mineral density was observed between the two groups
Nyrop et al., 2017	6-week randomized controlled trial of a home-based walking program	Self-reported joint symptoms and psychosocial measures	62 post-menopausal women diagnosed with stage 0-III breast cancer, with aromatase inhibitor-associated arthralgia	Intervention group participants reported significantly increased walking minutes per week, reduced stiffness, less difficulty with activities of daily living, and less perceived helplessness in managing joint symptoms
Valle et al., 2017	3-arm, 6-month randomized controlled trial of a physical activity intervention (activity tracker and tailored feedback based on objective weight; tailored feedback alone; or control)	Change in weight	35 African American breast cancer survivors, stage I-IIIa	Median weight change was −0.9 in the intervention group that included activity trackers vs. 0.2% gain in the control group
Westphal et al., 2018	48-week multi-center randomized controlled trial of counseling and unsupervised exercise training vs. supervised physical training (24 weeks) followed by unsupervised training (additional 24 weeks). The supervised training was comprised of 45 minutes of stationary cycling and 30 minutes of resistance training twice a week.	Maximum power output on a cycle ergometer after 24 weeks of exercise	42 early-stage breast cancer patients receiving aromatase inhibitor treatment	After 24 weeks, the supervised arm achieved a significantly higher maximum output in watt (mean 132 +/− SD 34, 95% CI 117-147) compared to baseline 107 +/−25, 95% CI 97–117, p=0.012) with a higher output than the unsupervised arm (115 +/− 25, 95% CI 105–125, P=0.059).
Hirschey et al.,2018	Randomized controlled trial of a home-based physical activity intervention (a booklet about physical activity for breast cancer survivors, that included narrative messages and writing and thinking exercises)	Multidimensional exercise outcome measure	60 breast cancer survivors, stage Ia-IIb	Subjective exercise (weekly minutes) increased 2 minutes, and objective exercise increased by 970 steps, every 4 weeks in the intervention group compared to the control group (p=0.2676 and p=0.0283, respectively)
Bail et al., 2018	12-month randomized controlled trial of a home-based mentored vegetable gardening intervention	Vegetable consumption, physical activity, performance and function, anthropometrics, biomarkers, quality-of-life	82 breast cancer survivors, stage 0-III	Compared with the controls, intervention participants reported significantly greater improvements in moderate physical activity and demonstrated improvements in the 2-minute step test and arm curl (p-values<0.05).
Lahart et al., 2018	6-month randomized controlled trial of a home-based physical activity intervention	Cardiorespiratory fitness and physical activity	32 breast cancer survivors who had completed adjuvant therapy	Magnitude-based inference analyses revealed at least small beneficial effects on absolute and relative *V*O_2_ max (cardiorespiratory fitness), and total and moderate physical activity in the intervention compared to the usual care group.

## References

[1] American Cancer Society. Cancer Facts & Figures 2019. Atlanta: American Cancer Society, 2019.

[2] Zhang X, Li Y and Liu D. Effects of exercise on the quality of life in breast cancer patients: a systematic review of randomized controlled trials. Supportive Care in Cancer, 2019, 27(1): 9–21. 10.1007/s00520-018-4363-2 30032399

[3] World Cancer Research Fund Report “Food, Nutrition, Physical Activity, and the Prevention of Cancer: a Global Perspective”. 2007,1(10): 464–469. 10.1007/s12082-007-0105-4

[4] Vance V, Mourtzakis M, McCargar L, Weight gain in breast cancer survivors: prevalence, pattern and health consequences. Obesity Reviews, 2011, 12(4): 282–94. 10.1111/j.1467-789X.2010.00805.x 20880127

[5] Rock CL and Demark-Wahnefried W. Nutrition and Survival after the Diagnosis of Breast Cancer: A Review of the Evidence. Journal of Clinical Oncology, 2002, 20(15): 3302–3316. 10.1200/JCO.2002.03.008 12149305 PMC1557657

[6] Knobf MT, Thompson SA, Fennie K, The Effect of a Community-Based Exercise Intervention on Symptoms and Quality of Life. Cancer Nursing, 2014, 37(2): E43–E50. 10.1097/NCC.0b013e318288d40e PMC3855633 23519041

[7] Smith SA, Ansa BE, Yoo W, Determinants of adherence to physical activity guidelines among overweight and obese African American breast cancer survivors: impliations for an intervention approach. Ethnicity & Health, 2018, 23(2): 194–206. 10.1080/13557858.2016.1256376 27838922 PMC5429994

[8] Coughlin SS and Smith SA. The insulin-like growth factor axis adipokines, physical activity, and obesity in relation to breast cancer incidence and recurrence. Cancer and Clinical Oncology, 2015, 4(2): 24–31. 10.5539/cco.v4n2p24 26251693 PMC4524449

[9] Schmidt T, van Mackelenbergh M, Wesche D, Physical activity influences the immune system of breast cancer patients. Journal of Cancer Research and Therapeutics, 2017, 13(3): 392–398. 10.4103/0973-1482.150356 28862198

[10] Pinto BM, Frierson M, Rabin C, Home-based physical activity intervention for breast cancer patients. Journal of Clinical Oncology, 2005, 23(15): 3577–3587. 10.1200/JCO.2005.03.080 15908668

[11] Wilson DB, Porter JS, Parker G, Anthropometric changes using a walking intervention in African American breast cancer survivors: a pilot study. Preventing Chronic Disease, 2005, 2(2): 1–7. https://www.cdc.gov/pcd/issues/2005/apr/04_0112.htm PMC1327710 15888227

[12] Vallance KH, Courneya KS, Plotnikoff RC, Randomized controlled trial of the effects of print materials and step pedometers on physical activity and quality of life in breast cancer survivors. Journal of Clinical Oncology, 2007, 25(17): 2352–2359. 10.1200/JCO.2006.07.9988 17557948

[13] Matthews CE, Wilcox S, Hanby CL, Evaluation of a 12-week home-based walking intervention for breast cancer survivors. Supportive Care in Cancer, 2007,15(2): 203–211. 10.1007/s00520-006-0122-x 17001492

[14] Yuen HK and Sword D. Home-based exercise to alleviate fatigue and improve functional capacity among breast cancer survivors. Journal of allied health, 2007, 36(4): e257–275. 19759996

[15] Payne JK, Held J, Thorpe J, Effect of exercise on biomarkers, fatigue, sleep disturbances, and depressive symptoms in older women with breast cancer receiving hormonal therapy. Oncology Nursing Forum, 2008, 35(4): 635–642. 10.1188/08.ONF.635-642 18591167

[16] Yang CY, Tsai JC, Huang YC, Effects of a home-based walking program on perceived symptom and mood status in post-operative breast cancer women receiving adjuvant chemotherapy. Journal of Advanced Nursing, 2011, 67(1): 158–168. 10.1111/j.1365-2648.2010.05492.x 20973811

[17] Kim SH, Shin MS, Lee HS, Randomized pilot test of a simultaneous stage-matched exercise and diet intervention for breast cancer survivors. Oncology Nursing Forum, 2011, 38(2): E97–E106. 10.1188/11.ONF.E97-E106 21356647

[18] Hatchett A, Hallam JS and Ford MA. Evaluation of a social cognitive theory-based email intervention designed to influence the physical activity of survivors of breast cancer. Psycho-Oncology, 2013, 22(4): 829–836. 10.1002/pon.3082 22573338

[19] Spector D, Deal AM, Amos KD, A pilot study of a home-based motivational exercise program for African American breast cancer survivors: clinical and quality-of-life outcomes. Integrative Cancer Therapies, 2014, 13(2): 121–132. 10.1177/1534735413503546 24105359 PMC10568972

[20] Denysschen CA, Burton H, Ademuyiwa F, Exercise intervention in breast cancer patients with aromatase inhibitor-associated arthralgia: a pilot study. European Journal of Cancer Care, 2014, 23(4): 493–501. 10.1111/ecc.12155 24289215

[21] Baruth M, Wilcox S, Der Ananian C, Effects of home-based walking on quality of life and fatigue outcomes in early stage breast cancer survivors a 12-week pilot study. Journal of Physical Activity and Health, 2015,12(s1): S110–S118. 10.1123/jpah.2012-0339 23963636

[22] Lahart IM, Metsios GS, Nevill AM, Randomized controlled trial of a home-based physical activity intervention in breast cancer survivors. BMC Cancer, 2016, 16: 234 10.1186/s12885-016-2258-5 26988367 PMC4797234

[23] Knobf MT, Jeon S, Smith B, Effect of a randomized controlled exercise trial on bone outcomes: influence of adjuvant endocrine therapy. Breast Cancer Research and Treatment, 2016, 155(3): 491–500. 10.1007/s10549-016-3693-3 26850265

[24] Nyrop KA, Callahan LF, Cleveland RJ, Randomized controlled trial of a home-based walking program to reduce moderate to severe aromatase inhibitor-associated arthralgia in breast cancer survivors. The Oncologist, 2017, 22(10): 1238–1248. 10.1634/theoncologist.2017-0174 28698390 PMC5634775

[25] Valle CG, Deal AM and Tate DF. Preventing weight gain in African American breast cancer survivors using smart scales and activity trackers: a randomized controlled pilot study. Journal of Cancer Survivorship, 2017, 11(1): 133–148. 10.1007/s11764-016-0571-2 27631874 PMC5269496

[26] Westphal T, Rinnerthaler G, Gampenrieder SP, Supervised versus autonomous exercise training in breast cancer patients: a multicenter randomized clinical trial. Cancer Medicine, 2018, 7(12): 5962–5972. 10.1002/cam4.1851 30415507 PMC6308077

[27] Hirschey R, Kimmick G, Hockenberry M, A randomized phase II trial of MOVING ON: An intervention to increase exercise outcome expectations among breast cancer survivors. Psycho-Oncology, 2018, 27(10): 2450–2457. 10.1002/pon.4849 30071146 PMC6684254

[28] Bail JR, Fruge AD, Cases MG, A home-based mentored vegetable gardening intervention demonstrates feasibility and improvements in physical activity and performance among breast cancer survivors. Cancer, 2018, 124(16): 3427–3435. 10.1002/cncr.31559 29932460 PMC6108896

[29] Lahart IM, Carmichael AR, Nevill AM, The effects of a home-based physical activity intervention on cardiorespiratory fitness in breast cancer survivors; a randomised controlled trial. Journal of Sports Sciences, 2018, 36(10): 1077–1086. 10.1080/02640414.2017.1356025 28745131

